# 

**Published:** 1860-08

**Authors:** 


### CONTENTS OF HALL'S JOURNAL OF HEALTH FOR AUGUST.

(New-York, $1 a year.)

Is Consumption Contagious? ... 169

Table Manners, ... 170

Tomatoes, ... 172

Ice Water, ... 173

Regimen, ... 174

Sleeping, ... 175

Tobacco-Users, ... 177

Cellars, ... 178

Spitting Blood, ... 179

Small Bed-chambers, ... 180

Each the Best, ... 181

Eight to Sixteen, ... 183

Drinking Water, ... 184

Drunken Women, ... 185

Metamorphoses, ... 187

Catarrh, ... 189

Living Yet, ... 190

